# Weight loss and metabolic benefits of bariatric surgery in China: A multicenter study

**DOI:** 10.1111/1753-0407.13430

**Published:** 2023-07-06

**Authors:** Wenhuan Feng, Zhiming Zhu, Xiaoying Li, Zhiguang Zhou, Shen Qu, Xitai Sun, Dalong Zhu

**Affiliations:** ^1^ Department of Endocrinology Drum Tower Hospital Affiliated with Nanjing University Medical School Nanjing China; ^2^ Department of Endocrinology, Daping Hospital Third Military Medical University Chongqing China; ^3^ Department of Endocrinology Zhongshan Hospital affiliated with Fudan University Shanghai China; ^4^ Department of Endocrinology The Second Xiangya Hospital of Central South University Changsha China; ^5^ Department of Endocrinology, The Tenth People's Hospital of Tongji University Shanghai China; ^6^ Department of General Surgery Drum Tower Hospital Affiliated with Nanjing University Medical School Nanjing China

**Keywords:** bariatric surgery, China, obesity, retrospective study, 减重手术, 中国, 肥胖症, 回顾性研究

## Abstract

**Background:**

This retrospective multicenter study evaluated the efficacy and safety of bariatric surgery in Chinese patients with obesity.

**Methods:**

Patients with obesity who underwent laparoscopic sleeve gastrectomy or laparoscopic Roux‐en‐Y gastric bypass and completed a 12‐month follow‐up between February 2011 and November 2019 were enrolled. Weight loss, glycemic and metabolic control, insulin resistance, cardiovascular risk, and surgery‐related complications at 12 months were analyzed.

**Results:**

We enrolled 356 patients aged 34.3 ± 0.6 years with a mean body mass index of 39.4 ± 0.4 kg/m^2^. Successful weight loss occurred in 54.6%, 86.8%, and 92.7% of patients at 3, 6, and 12 months, respectively, with no difference in percent excess weight loss between the laparoscopic sleeve gastrectomy and laparoscopic Roux‐en‐Y gastric bypass surgery groups. The average percentage of total weight loss was 29.5% ± 0.6% at 12 months; 99.4%, 86.8%, and 43.5% of patients achieved at least 10%, 20%, and 30% weight loss, respectively, at 12 months. Significant improvements in metabolic indices, insulin resistance, and inflammation biomarkers were observed at 12 months.

**Conclusions:**

Bariatric surgery resulted in successful weight loss and improved metabolic control, insulin resistance, and cardiovascular risk in Chinese patients with obesity. Both laparoscopic sleeve gastrectomy and laparoscopic Roux‐en‐Y gastric bypass are suitable approaches for such patients.

## Graphical abstract

Highlights

Laparoscopic sleeve gastrectomy and laparoscopic Roux‐en‐Y gastric bypass are effective for reducing weight and improving metabolic parameters in Chinese patients with obesity. These interventions resulted in improved glycemic control, as evidenced by decreased glycated hemoglobin levels, reduced insulin resistance, and improved cardiovascular profiles.

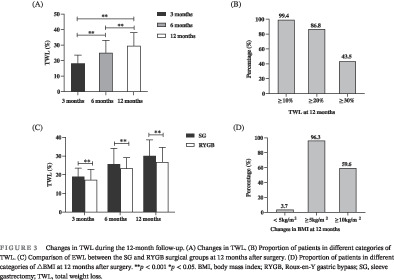

## 摘要

背景:本回顾性多中心研究评估了减重手术在中国肥胖患者中的有效性和安全性。

方法:研究纳入了在2011年2月至2019年11月期间接受腹腔镜袖状胃切除术或腹腔镜Roux‐en‐Y胃旁路术并完成12个月随访的肥胖患者。分析12个月时体重减轻, 血糖和代谢控制, 胰岛素抵抗, 心血管风险和手术相关并发症。

结果:共纳入356例患者, 年龄(34.3±0.6)岁, 体重指数(39.4±0.4)kg/m^2^。3个月, 6个月和12个月时, 分别有54.6%, 86.8%和92.7%的患者成功减重, 腹腔镜袖状胃切除术组和腹腔镜Roux‐en‐Y胃旁路术组的额外减重百分比无差异。12个月时平均总体重下降百分比为29.5%±0.6%, 12个月时, 分别有99.4%, 86.8%和43.5%的患者体重减轻至少10%, 20%和30%。12个月时观察到代谢指标, 胰岛素抵抗和炎症生物标志物显著改善。

结论:减重手术可成功减轻中国肥胖患者的体重, 改善代谢控制, 胰岛素抵抗和心血管风险。腹腔镜袖状胃切除术和腹腔镜Roux‐en‐Y胃旁路术均适用于此类患者。

## 1 INTRODUCTION

As the prevalence of obesity has dramatically increased in the past 30 years, China has become the country with the largest population with obesity worldwide.^1^ Obesity is associated with several comorbidities, including type 2 diabetes, dyslipidemia, hypertension, nonalcoholic fatty liver disease (NAFLD), infertility, cardiovascular diseases, osteoarthritis, and some cancers, and represents a significant public health burden.^2^, ^3^, ^4^, ^5^

Bariatric surgery is recommended for patients in Western countries and China with severe or moderate obesity and metabolic disorders in whom sustained weight loss cannot be achieved through lifestyle modification and pharmacotherapy.^6^, ^7^, ^8^ Although the number of patients who have undergone bariatric surgery has increased in mainland China in recent years, the proportion of patients with obesity undergoing bariatric surgery in China is far lower than that in Western countries.^9^, ^10^, ^11^, ^12^, ^13^ This may be owing to insufficient evidence to support the benefits of weight loss through bariatric surgery in this population and because the procedure is not covered by medical insurance in China. Some single‐center and multicenter studies have confirmed the beneficial effects of bariatric surgery on maintaining sustained weight loss and alleviating or reversing obesity‐related complications in mainland China.^9^, ^10^, ^11^, ^12^, ^13^ However, few studies with larger sample sizes with multicenter follow‐up have been conducted to clarify the benefits and risks of bariatric surgery in this population and help multidisciplinary bariatric surgery teams make informed decisions. Additionally, the follow‐up status of patients with obesity undergoing bariatric surgery in mainland China must be clarified to help improve the quality of follow‐up in the future.

To address these concerns, we performed a retrospective multicenter observational study in five publicly funded tertiary comprehensive hospitals in mainland China to clarify the efficacy of bariatric surgery in patients with obesity.

## 2 METHODS

### 2.1 Study design

This retrospective multicenter study included patients with obesity who underwent laparoscopy sleeve gastrectomy (SG) or laparoscopy Roux‐en‐Y gastric bypass (RYGB) in the general surgery departments of five publicly funded tertiary comprehensive hospitals in mainland China, from February 2011 to November 2019. These included Drum Tower Hospital, Nanjing University Medical School; Daping Hospital, Third Military Medical University; Zhongshan Hospital, affiliated with Fudan University; the Tenth People's Hospital of Tongji University; and the Second Xiangya Hospital of Central South University. Endoscopic surgeons in the Tenth People's Hospital of Tongji University and Zhongshan Hospital Affiliated to Fudan University mostly preferred SG, and endoscopic surgeons in the other three hospitals often preferred RYBG for patients with type 2 diabetes mellitus and SG for other patients (Table S1). Patients from the Tenth People's Hospital of Tongji University were not required to return to the hospital for a medical visit at 1 month after the operation, and thus only 3‐, 6‐, and 12‐month follow‐up data from all hospitals were analyzed. The patient data were retrieved from electronic charts. A multidisciplinary team including diabetologists, endoscopic surgeons, diabetes nurse educators, dietitians, exercise physiologists, psychiatrists, and patient case managers participated in the preoperative and postoperative care of the patients. After bariatric surgery, patients were administered vitamin D and calcium tablets, as well as other multivitamins, based on the guidelines.^8^, ^14^ In addition to routine surgical care, pre‐ and postbariatric care included (a) psychological comfort for patients, to avoid anxiety and fear of bariatric surgery; (b) guidance on drinking water, diet, medication, and walking during hospitalization, especially early after surgery; and (c) timely communication with the patients around the time of admission and postoperative follow‐up via phone or WeChat. The study was approved by the ethics committees of all hospitals, and the anonymity of each patient was preserved. All participants provided informed consent before the study. The study conformed to the provisions of the Declaration of Helsinki.

### 2.2 Surgical participants

Three hundred and sixty‐five patients (232 in the SG group and 124 in the RYBG group) aged 15–69 years with a mean baseline body mass index (BMI) of 39.4 ± 0.4 kg/m^2^ underwent bariatric surgery. Type 2 diabetes was diagnosed based on the 2017 Chinese Diabetes Society diagnostic criteria, defined as symptoms of diabetes and fasting blood glucose (FBG) ≥7.0 mmol/L and/or glucose level ≥11.1 mmol/L at 120 min during the 75‐g glucose tolerance test, or patients with history of anti‐diabetic drug use.^8^ Hypertension was defined as a systolic blood pressure (SBP) ≥140 mm Hg, diastolic blood pressure (DBP) ≥90 mm Hg, or administration of antihypertensive drug therapy.^15^ Hyperuricemia was defined as a serum uric acid level ≥420 mmol/L and/or administration of antihyperuricemia medicines.^16^ Dyslipidemia was defined as a fasting high‐density lipoprotein cholesterol (HDL‐C) level <1.03 mmol/L, and/or triglycerides ≥1.70 mmol/L, and/or low‐density lipoprotein cholesterol (LDL‐C) ≥2.6 mmol/L, and/or total lipoprotein cholesterol ≥5.2 mmol/L, or the use of lipid‐lowering drugs.^15^ Gastroesophageal reflux and *H. pylori* infection were diagnosed via gastroscopy before bariatric surgery. *H. pylori* infection was usually not treated preoperatively because the patients often underwent metabolic surgery 3–5 days after hospitalization, after completing the preoperative examination. Information on history of stroke, coronary heart disease, heart failure, and limb venous thrombosis was retrieved from the patients' clinical records. Obstructive sleep apnea was diagnosed based on the patients' medical history and/or preoperative polysomnography findings. NAFLD was defined based on ultrasonographic features and the absence of secondary causes of fatty liver, such as history of alcohol consumption ≥140 g and ≥210 g per week for women and men, respectively, or history of viral hepatitis.^5^

Bariatric surgery was not recommended for patients with the following conditions: type 1 diabetes, type 2 diabetes with poor pancreatic β‐cell function (fasting serum C‐peptide ≥135 pmol/L) or severe diabetes complications, drug or alcohol addiction, unmanaged mental illness, cerebrovascular disease or myocardial infarction in the previous 6 months, contraindications for receiving bariatric surgery, and pregnancy or plans for pregnancy in female participants.^8^, ^14^

### 2.3 Primary and secondary outcomes

The primary outcomes were successful weight loss, defined as the percentage of excess weight loss (EWL) = (initial BMI − BMI at follow‐up point)/(initial BMI − 25) >50% at 3, 6, and 12 months after the operation, as well as the difference in EWL between the SG and RYGB surgical groups.^15^ The secondary outcomes were the percent of total weight loss (TWL), BMI decrease, blood pressure, glycemic control, lipid profile, serum uric acid level, liver and renal function, and homeostasis model assessment of insulin resistance (HOMA‐IR) (defined as fasting insulin × FBG/22.5), measured at the 3‐, 6‐, and 12‐month follow‐up points.^17^

### 2.4 Statistical analysis

An independent statistician conducted all statistical analyses using SPSS version 25.0 (IBM Corp., Armonk, NY, USA). Two‐tailed *p* values of <.05 were considered statistically significant. Categorical data are described as frequencies and were analyzed using the chi‐square test. Quantitative variables are presented as the mean ± SD. The Mann–Whitney *U*‐test was performed to analyze continuous variables. One‐way repeated measures analysis of variance was used to evaluate quantitative variables within the same group. Differences between the treatment groups were determined using analysis of covariance after adjusting for baseline values.

## 3 RESULTS

**FIGURE 1:**
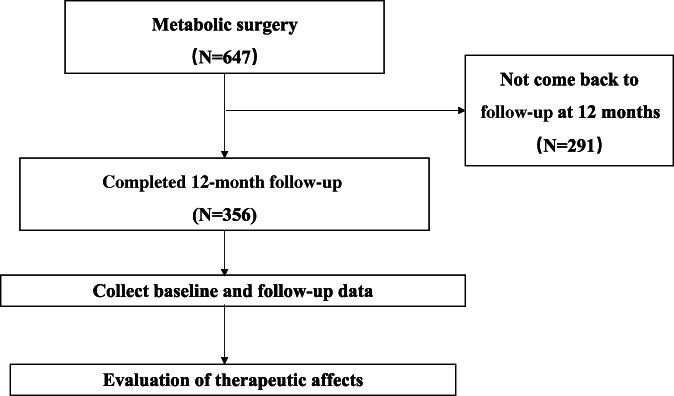
Flow chart of the study participants.

In total, 647 patients were included in this study. The data of 356 patients (167 men and 189 women) who completed the 12‐month follow‐up were available for the final evaluation (Figure 1), and the baseline obesity‐related comorbidities and clinical characteristics of these patients are shown in Tables 1 and S2, respectively.

**TABLE 1:** Characteristics of the patients at baseline and at 12 months.

Characteristic	N	Baseline	12 months	d	*p*
Number (n)	‐	356	‐	‐	‐
Age (years)	356	34.3 ± 11.1	‐	‐	‐
Sex (Man/Woman)	356	167/189	‐	‐	‐
Weight (kg)	356	111.3 ± 23.9	78.2 ± 15.5	33.0 ± 14.6	< 0.001
BMI (kg/m^2^)	356	39.4 ± 6.9	27.7 ± 4.4	11.7 ± 4.9	< 0.001
BMI < 24 kg/m^2^	‐	0%	21.6%	−21.6%	< 0.001
Waist circumference (cm)	233	119.6 ± 15.4	93.2 ± 12.1	26.3 ± 11.4	< 0.001
SBP (mmHg)	212	135.9 ± 19.3	119.6 ± 15.4	16.3 ± 19.3	< 0.001
SBP < 130 mmHg	‐	40.6%	75.5%	−34.9%	< 0.001
DBP (mmHg)	212	85.2 ± 13.5	75.0 ± 11.5	10.2 ± 14.9	< 0.001
HbA1c (%)	299	6.8 ± 1.8	5.4 ± 0.5	1.5 ± 1.7	< 0.001
HbA1c < 7%	‐	65.9%	98.7%	−32.8%	< 0.001
HbA1c < 6%	‐	44.8%	91.0%	−46.2%	< 0.001
FBG (mmol/L)	321	7.0 ± 2.9	4.7 ± 0.8	2.3 ± 2.8	< 0.001
FBG <5.6 mmol/L	‐	41.7%	89.4%	−47.7%	< 0.001
120 min glucose (mmol/L)	280	10.9 ± 5.1	4.8 ± 1.9	6.1 ± 5.2	< 0.001
Fasting insulin (μU/mL)	233	30.9 ± 20.6	9.8 ± 5.7	21.1 ± 20.0	< 0.001
120 min insulin (μU/mL)	197	144.6 ± 119.4	27.1 ± 41.2	117.5 ± 127.1	< 0.001
HOMA‐IR (mmol/L, μU/mL)	232	9.8 ± 9.4	2.1 ± 1.6	7.7 ± 9.2	< 0.001
HOMA‐IR <1.45	‐	0.4%	35.3%	−34.9%	< 0.001
TG (mmol/L)	315	2.3 ± 1.9	1.0 ± 0.5	1.3 ± 1.8	< 0.001
TC (mmol/L)	314	4.6 ± 1.0	4.3 ± 0.9	0.3 ± 1.0	< 0.001
HDL‐C (mmol/L)	307	1.0 ± 0.2	1.4 ± 0.3	−0.4 ± 0.3	< 0.001
LDL‐C (mmol/L)	314	2.8 ± 0.8	2.5 ± 0.8	0.3 ± 0.9	< 0.001
LDL‐C < 2.6 mmol/L	‐	42.4%	58.6%	−16.2%	< 0.001
ALT (U/L)	303	57.9 ± 47.6	17.8 ± 11.6	40.0 ± 48.2	< 0.001
AST (U/L)	302	38.5 ± 38.8	18.5 ± 7.9	20.0 ± 38.9	< 0.001
rGT (U/L)	260	51.0 ± 37.1	18.0 ± 11.9	32.9 ± 32.5	< 0.001
Cr (umol/L)	292	60.0 ± 16.6	59.1 ± 14.6	0.8 ± 10.1	0.179
UA (umol/L)	301	419.9 ± 98.7	347.5 ± 90.2	72.4 ± 84.9	< 0.001

*Note*: *p* values of < 0.05 were considered significant. Quantitative Variables are presented as the mean ± SD.

Abbreviations: ALT, serum alanine aminotransferase; AST, aspartate aminotransferase; BMI, body mass index; Cr, creatinine; DBP, diastolic blood pressure; FBG, fasting blood glucose; GGT, glutamyltrans peptidase; HbA1c, glycated hemoglobin; HDL‐C, high‐density lipoprotein cholesterol; HOMA‐IR, homeostasis model assessment of insulin resistance; LDL‐C, low‐density lipoprotein cholesterol; SBP, systolic blood pressure; TC; total lipoprotein cholesterol; TG, triglycerides; UA, uric acid.

### 3.1 Primary outcome

**FIGURE 2:**
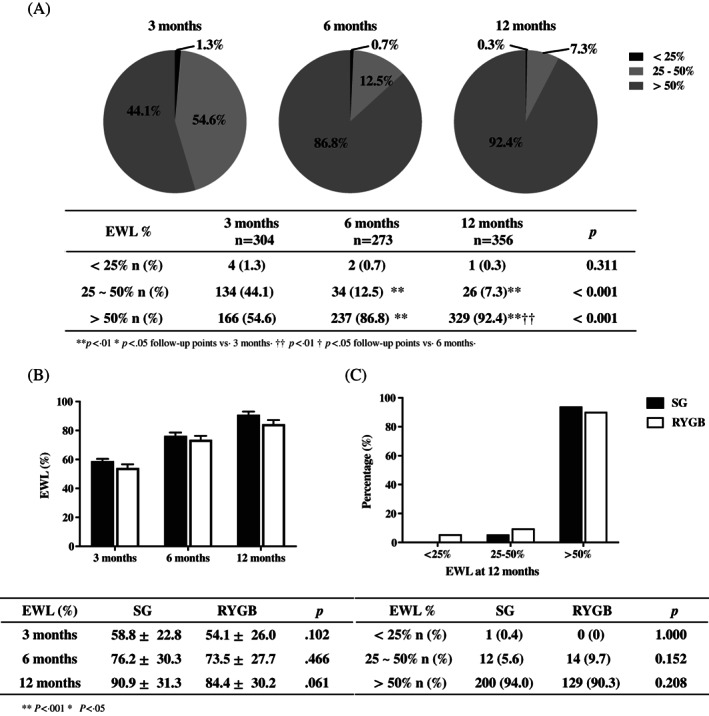
Changes in EWL during the 12‐month follow‐up. (A) Proportion of patients at different categories of EWL at 3, 6, and 12 months after surgery. (B) Comparison of EWL between the SG and RYGB surgical groups at 3, 6, and 12 months after surgery. (C) Comparison of the proportion of patients in different categories of EWL between the SG and RYGB surgical groups at 12 months after surgery. **p < 0.001 vs 3 months. †† vs 6 months. EWL, excess weight loss; RYGB, Roux‐en‐Y gastric bypass; SG, sleeve gastrectomy.

The percentage of patients who experienced successful weight loss, defined as EWL >50%, gradually and significantly increased over time, with values of 54.6%, 86.8%, and 92.7% at 3, 6, and 12 months, respectively (Figure 2A). There was no difference in EWL between the surgical groups at the same follow‐up times (*p* = 0.061–0.466) (Figure 2B) or in the different levels of percentage of EWL (<25%, *p* = 1.000; 25–50%, *p* = 0.152; >50%, *p* = 0.208) (Figure 2C).

### 3.2 TWL, BMI, and waist circumference

**FIGURE 3:**
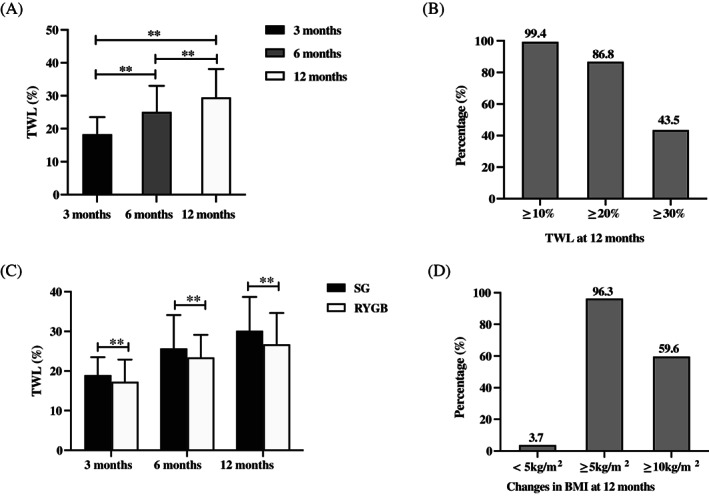
Changes in TWL during the 12‐month follow‐up. (A) Changes in TWL. (B) Proportion of patients in different categories of TWL. (C) Comparison of EWL between the SG and RYGB surgical groups at 12 months after surgery. (D) Proportion of patients in different categories of △BMI at 12 months after surgery. **p < 0.001 *p < 0.05. BMI, body mass index; RYGB, Roux‐en‐Y gastric bypass; SG, sleeve gastrectomy; TWL, total weight loss.

The average TWL of all patients was increased after metabolic surgery by 18.3% ± 5.2%, 25.1% ± 7.9%, and 29.5% ± 8.6% at 3, 6, and 12 months, respectively (*p* < 0.001) (Figure 3A); 99.4%, 86.8%, and 43.5% of patients achieved a TWL greater than or equal to 10%, 20%, and 30%, respectively, at 12 months (Figure 3B). Patients who underwent SG had greater TWL than that of those who underwent RYGB at the same follow‐up visits (all *p* < 0.001) (Figure 3C).

We found that 96.3% and 59.6% of patients showed decreases in their BMI of at least 5 and 10 kg/m^2^, respectively, and 21.6% of patients achieved a BMI of <24 kg/m^2^ 12 months after surgery (Figure 3D, Table 1). Patients who underwent SG exhibited greater decrease in BMI than did those who underwent RYGB (absolute difference, 1.4 kg/m^2^; 95% confidence interval [CI], 0.3–2.4 kg/m^2^; *p* = 0.009), whereas similar percentages of patients in both surgical groups attained a BMI of <24 kg/m^2^ at 12 months after operation (*p* = 0.065) (Table 2).

**TABLE 2:** Characteristics of the patients in the SG and RYBG groups at baseline and at 12 months.

Characteristic	SG		RYGB						
	N	Baseline	1 year	No.	Baseline	1 year	*p* baseline	Estimated treatment difference, SG vs. RYGB mean (95% CI)	*p* Decreased value between two groups
Number (n)	‐	232	‐	‐	124	‐	‐	‐	‐
Sex (Man/Woman))	‐	113/119	‐	‐	54/70	‐	0.353	‐	‐
Age (years)	‐	32.7 ± 10.9	‐	‐	37.2 ± 10.9	‐	< 0.001	‐	‐
Weight (kg)	232	112.9 ± 24.2	78.1 ± 15.6**	124	108.2 ± 23.0	78.5 ± 15.3**	0.072	5.2 (2.0 to 8.3)	0.001
BMI (kg/m^2^)	232	39.5 ± 6.8	27.4 ± 4.4**	124	39.1 ± 7.2	28.4 ± 4.4**	0.616	1.4 (0.3 to 2.4)	0.009
BMI < 24 kg/m^2^	‐	0%	24.6%**	‐	0%	16.1%**	‐	‐	0.065
Waist circumference (cm)	151	119.8 ± 14.4	92.1 ± 11.4**	82	119.1 ± 17.3	95.3 ± 13.2**	0.723	3.9 (0.9 to7.0)	0.011
SBP (mmHg)	152	135.3 ± 18.5	119.0 ± 15.1**	60	137.2 ± 21.1	120.9 ± 16.3**	0.514	−0.0 (−5.9 to 5.8)	0.988
SBP < 130 mmHg	‐	42.8%	77.0%**	‐	35.0%	71.7%**	0.300	‐	0.723
DBP (mmHg)	152	84.6 ± 13.5	74.3 ± 11.6**	60	86.6 ± 13.4	76.7 ± 11.3**	0.325	0.4 (−4.1 to 4.8)	0.873
HbA1c (%)	197	6.5 ± 1.5	5.3 ± 0.5**	102	7.5 ± 2.1	5.5 ± 0.6**	< 0.001	−0.8 (−1.2 to −0.4)	<0.001
HbA1c < 7%	‐	73.6%	98.5%**		51.0%	99.0%**	< 0.001	‐	0.618
HbA1c < 6%	‐	53.8%	94.4%**	‐	27.5%	84.3%**	< 0.001	‐	0.100
FBG (mmol/L)	210	6.6 ± 2.5	4.5 ± 0.6**	111	7.9 ± 3.5	5.1 ± 1.0**	< 0.001	−0.8 (−1.5 to −0.1)	0.025
FBG <5.6 mmol/L	‐	46.7%	94.8%**	‐	32.4%	79.3%**	0.014	‐	<0.001
120 min glucose (mmol/L)	187	10.2 ± 4.6	4.6 ± 1.7**	93	12.4 ± 5.7	5.2 ± 2.3**	0.001	−1.7 (−3.1 to −0.3)	0.019
Fasting insulin (uU/mL)	147	32.7 ± 21.7	9.5 ± 5.5**	86	27.9 ± 18.3	10.3 ± 6.1**	0.087	5.5 (0.2 to 10.8)	0.041
120 min insulin (uU/mL)	126	163.2 ± 132.3	32.9 ± 48.6**	71	111.4 ± 83.0	16.8 ± 18.9**	0.001	35.8 (3.2 to 68.3)	0.058
HOMA‐IR (mmol/L, uU/mL)	147	10.0 ± 9.1	2.0 ± 1.2**	85	9.6 ± 10.0	2.4 ± 2.1**	0.762	0.8 (−1.7 to 3.2)	0.539
HOMA‐IR <1.45	‐	0.7%	39.5%**	‐	0%	28.2%**	1.000	‐	0.097
TG (mmol/L)	204	2.1 ± 1.3	1.0 ± 0.5**	111	2.8 ± 2.6	1.1 ± 0.5**	0.008	−0.6 (−1.1 to −0.1)	0.022
TC (mmol/L)	203	4.5 ± 1.0	4.4 ± 0.9	111	4.8 ± 1.1	4.1 ± 0.8**	0.020	−0.6 (−0.9 to −0.4)	<0.001
HDL‐C (mmol/L)	201	1.0 ± 0.2	1.4 ± 0.3**	107	1.0 ± 0.3	1.3 ± 0.3**	0.219	−0.1 (−0.1 to −0.0)	0.032
LDL‐C (mmol/L)	205	2.9 ± 0.8	2.7 ± 0.9**	109	2.7 ± 0.9	2.2 ± 0.6**	0.283	−0.3 (−0.5 to −0.1)	0.003
LDL‐C < 2.6 mmol/L	‐	40.5%	52.2%**	‐	45.9%	70.6%**	0.358	‐	<0.001
ALT (U/L)	192	61.8 ± 50.1	14.0 ± 9.8**	111	51.1 ± 42.3	24.4 ± 11.5**	0.050	21.0 (9.9 to 32.0)	<0.001
AST (U/L)	192	37.9 ± 25.6	16.5 ± 6.2**	110	39.6 ± 54.7	22.1 ± 9.0**	0.716	3.9 (−5.3 to 13.1)	0.403
GGT (U/L)	173	50.1 ± 37.7	17.0 ± 11.3**	87	51.2 ± 36.2	20.1 ± 12.7**	0.953	2.9 (−5.6 to 11.3)	0.505
Cr (umol/L)	186	60.3 ± 15.5	60.2 ± 15.0	106	59.2 ± 18.5	57.2 ± 13.8	0.587	−1.9 (−4.3 to 0.5)	0.122
UA (umol/L)	192	426.3 ± 92.9	353.5 ± 91.9**	109	408.5 ± 107.7	338.6 ± 86.8**	0.131	3.9 (−16.2 to 24.0)	0.703

*Note*: **p < 0.01 *p < 0.05 baseline vs 1 year. *p* values of < 0.05 were considered significant. Quantitative variables are presented as the mean ± SD.

Abbreviations: RYGB, laparoscopy Roux‐en‐Y gastric bypass; SG, sleeve gastrectomy.

Waist circumference significantly decreased in all patients (△ = 26.3 ± 11.4 cm; *p* < 0.001), with greater reductions observed following SG compared with those following RYGB (*p* = 0.011) at 12 months after surgery (Tables 1 and 2).

### 3.3 Glycemic control and insulin resistance

Before surgery, 143 (40.2%) and 100 (28.1%) patients had type 2 diabetes and impaired glucose regulation, respectively (Table S2). At 12 months after surgery, glycemic control (FBG, 120‐min serum glucose, glycated hemoglobin [HbA1c], and percentage of patients with FBG <5.6 mmol/L, HbA1c <6%, and HbA1c <7%) was markedly improved in all patients (all *p* < 0.001) (Table 1). In contrast to the SG group, a higher rate of type 2 diabetes (52.4% vs 33.6%, *p* < 0.001); higher levels of FBG, 120‐min glucose, and HbA1c; and lower percentages of FBG <5.6 mmol/L and HbA1c <7% and <6% at baseline (*p* < ‐0.001–*p* = 0.014) were noted in the RYGB group (Tables 2 and S2). Additionally, a lower percentage of patients showed FBG <5.6 mmol/L at 12 months after surgery (*p* < 0.001–*p* = 0.025) (Table 2). There was no difference in the percentages of patients who attained HbA1c <6% (*p* = 0.100) and <7% (*p* = 0.618) between the surgical groups (*p* = 0.474) (Table 2).

Metabolic surgery led to a significant decrease in the HOMA‐IR (△ = 7.7 ± 9.2 mmol/L, μU/mL), and a greater number of patients who underwent surgery had a HOMA‐IR <1.45 mmol/L, μU/mL (both, *p* < 0.001) (Table 1). Similar reductions in the HOMA‐IR and the percentage of patients with a HOMA‐IR <1.45 mmol/L, μU/mL were observed in the two surgical groups at 12 months after operation (*p* = 0.097–0.539) (Table 2).

### 3.4 Blood pressure

At the time of surgery, 217 (61.0%) patients had hypertension (Table S2). SBP and DBP improved in all patients, and a greater number of patients had SBP <130 mm Hg at 12 months after surgery (all *p* < 0.001 vs respective baselines) (Table 1). The reductions in SBP and DBP were similar in the SG and RYGB surgical groups at 12 months (*p* = 0.988 and 0.873, respectively) (Table 2).

### 3.5 Serum lipid and uric acid levels

At baseline, 346 (97.2%) patients were diagnosed with dyslipidemia (Table S2). Significant amelioration of serum levels of triglycerides, total cholesterol, HDL‐C, and LDL‐C was observed (all *p* < 0.001 vs respective baselines), and a greater number of patients attained an LDL‐C of <2.6 mmol/L at 12 months after surgery (*p* < 0.001 vs baseline) (Table 1). The RYGB group showed higher serum levels of triglycerides and total cholesterol (*p* = 0.008–0.020) than did the SG group at baseline. At 12 months after operation, there were greater reductions in the serum levels of triglycerides, total cholesterol, and LDL‐C and a larger percentage of patients had LDL <2.6 mmol/L in the RYGB group (*p* < 0.001–0.022), whereas greater increases in the serum levels of HDL‐C occurred in the SG group (*p* = 0.032) (Table 2).

Before surgery, 171 (48.0%) of the 356 patients had hyperuricemia (Table S2). Metabolic surgery resulted in notably reduced serum uric acid levels (△ = 72.4 ± 84.9 μmol/L; *p* < .001) (Table 1), with similar reductions observed between the two surgical groups (*p* = 0.703) (Table 2).

### 3.6 Liver outcomes

At the time of surgery, 194 (54.5%) patients had NAFLD (Table S2). At 12 months following surgery, liver function was significantly improved in all patients (all *p* < 0.001) (Table 1). There was no difference in serum aspartate aminotransferase (AST) and gamma‐glutamyl transferase levels at baseline and at 12 months after the operation between the SG group and RYGB group (*p* = 0.050–0.953), but there was greater reduction in the levels of serum alanine aminotransferase at 12 months after surgery in the SG group (*p* < 0.001 vs baseline) (Table 2).

### 3.7 Inflammation and nutrition biomarkers

Compared with baseline, all patients experienced significant reduction in the inflammatory biomarker C‐reactive protein, white blood cell count, and neutrophils (all *p* < 0.001) at 12 months after surgery (Table S3). Regarding nutrition biomarkers, we observed significant reductions in hemoglobin and vitamin B12 (*p* < 0.001 and *p* = 0.001, respectively), and significant increases in folic acid and 25‐hydroxyvitamin D (*p* = 0.005 and *p* < 0.001, respectively) at 12 months after surgery (Table S3). Compared with men, women had lower hemoglobin and higher folic acid levels (*p* < 0.001 and *p* = 0.001, respectively) at baseline and greater reduction in hemoglobin (*p* = 0.002) 12 months postoperatively (Table S4). Patients who underwent SG had a greater increase in 25‐hydroxyvitamin D levels (*p* < 0.001) 12 months postoperatively. Patients who underwent RYBG had higher folic acid and lower vitamin B12 levels (*p* = 0.002 and *p* = 0.013, respectively) 12 months postoperatively (Table S5).

### 3.8 SG and RYBG in patients with and without type 2 diabetes

The patients were divided into two groups according to the presence of type 2 diabetes. Among those with type 2 diabetes, greater weight loss and greater reduction of BMI, waist circumference, serum FBG, 120 min glucose, serum alanine aminotransferase (ALT) and AST levels were found in the SG group (*p* < 0.01–0.05). More patients in the RYGB group had an FBG <5.6 mmol/L, LDL‐C < 2.6 mmol/L (*p* < 0.01–0.05), and serum total cholesterol (TC) and LDL‐C levels were lower in the RYGB group (both *p* < 0.01) (Table S8). In contrast, among those without type 2 diabetes, the decrease in body weight, BMI, and waist circumference were similar between the surgical groups. The patients in the SG group had lower DBP, serum FBG, ALT, and AST levels but higher serum 120 min insulin levels (*p* < 0.01–0.05). However, the patients in the RYGB group had lower serum TC, LDL‐C, and uric acid levels (*p* < 0.01–0.05) (Table S9).

### 3.9 Follow‐up rate and adverse events

Over time, the proportion of patients who returned to their respective operation hospitals for follow‐up visits gradually decreased; the follow‐up rates were lower in two of the included hospitals and slightly lower in the patients who underwent RYGB (Figures S1 and S2). The patients who returned for follow‐up visits had higher BMI and waist circumference (*p* = 0.001 and *p* = 0.047, respectively), and higher rates of impaired glucose regulation, hypertension, dyslipidemia, NAFLD, and *H. pylori* infection (*p* < 0.001–0.040) than those who did not return for follow‐up (Tables S6 and S7). Data regarding adverse events during the follow‐up of patients in the Tenth People's Hospital of Tongji University were not reported. Among the other institutions, there were no significant differences in adverse events for up to 12 months following SG and RYGB, except for a higher incidence of dumping syndrome in the RYGB group (*p* = 0.020) and a greater incidence of cholecystitis in the SG group (*p* = 0.004) (Table S10). None of the patients died during the follow‐up.

## 4 DISCUSSION

In this multicenter retrospective study of Chinese patients with moderate to severe obesity, most (92.7%) patients attained successful weight loss (defined as EWL >50%) at 12 months after metabolic surgery, accompanied by remarkable improvements in blood pressure, glucolipid metabolism, insulin resistance, liver function, and serum levels of uric acid and inflammatory biomarkers. Directionally consistent and qualitatively similar weight loss and alleviation of the metabolic markers were observed in both the SG and RYGB surgical groups.

Metabolic surgery is recommended for patients with severe/moderate obesity because greater weight loss can be achieved compared with that resulting from therapeutic measures.^6^, ^7^, ^8^, ^9^, ^10^, ^11^, ^12^, ^18^, ^19^ Consistent with the results of previous studies in Western countries, the majority of patients achieved successful weight loss in a short period of time, with most patients achieving successful weight loss by 12 months after surgery.^18^, ^19^, ^20^, ^21^ Additionally, 99.4% of patients lost at least 10% of their body weight compared with baseline, with an average weight loss of almost 30%. In nearly 60% of patients, BMI was reduced by at least 10 kg/m^2^, and 21.6% of patients had reached a normal weight by the 12‐month follow‐up, based on the diagnostic criteria for the Chinese population.^22^ Previous studies have suggested that bariatric surgery is a reasonable choice for individuals with moderate to severe obesity who must lose ≥20% of their weight,^18^, ^19^, ^20^, ^21^ and pharmacotherapy and behavioral modification are recommended for patients with mild obesity who need to lose only 5%–15% of their body weight.^23^, ^24^, ^25^, ^26^, ^27^ Successful weight loss following metabolic surgery in Chinese patients with obesity may explain why the number of metabolic surgeries has rapidly increased in mainland China in recent years.^9^, ^10^, ^11^, ^12^, ^13^

This study confirmed the higher prevalence (28.1%–97.2%) of obesity‐related comorbidities, including hypertension, type 2 diabetes and impaired glucose regulation, dyslipidemia, hyperuricemia, and NAFLD, in patients with obesity compared with their prevalence in the general Chinese population (12.8%–46.4%) and worldwide (9.8%–31.1%), as reported previously.^9^, ^10^, ^11^, ^12^, ^13^, ^18^, ^19^, ^20^, ^21^, ^28^, ^29^, ^30^ These diseases comprise important risk factors for atherosclerotic diseases, such as coronary atherosclerosis and stroke.^6^ Although the average age of patients in the present study was only 34.8 years, several patients had experienced coronary atherosclerosis and stroke before surgery, confirming that the risk of these diseases is higher in patients with obesity than that in those without obesity.^6^ Similar to other studies, we found that metabolic surgery led to improvements in blood pressure, glucolipid metabolism, and blood transaminase and uric acid levels, and reduced the insulin resistance index and levels of inflammatory biomarkers by the 12‐month follow‐up, which may help reduce future risk of atherosclerotic diseases.^9^, ^10^, ^11^, ^12^, ^13^, ^18^, ^19^ In long‐term retrospective observational studies of metabolic surgery (mean follow‐up of 7–10.9 years), metabolic surgery resulted in remarkable greater reductions in the rates of myocardial infarction and cardiovascular death compared with nonsurgical interventions, confirming the long‐term benefit of rapidly and effectively decreasing cardiovascular risk after metabolic surgery.^20^, ^21^, ^31^, ^32^ Metabolic control was improved after surgery, with a large increase in the percentage of successful weight loss, from 54.6% at 3 months to 92.7% at 12 months, reconfirming that this approach is effective for weight control in patients with moderate to severe obesity and metabolic disorders.

Diagnostic and treatment guidelines for obesity and type 2 diabetes recommend weight loss of 5%–10% within 3–6 months to reduce obesity‐related complications.^8^ However, the present study and in other metabolic surgery studies indicated that weight loss of >20%–30% is a more appropriate target for patients with moderate to severe obesity.^9^, ^10^, ^11^, ^12^, ^13^, ^18^, ^19^ Greater weight loss would produce greater benefit comorbidity resolution in obese patients with or without metabolic surgery.^33^, ^34^ Thus, it may be necessary to introduce quantitative weight loss targets based on different BMI values for patients with obesity, similar to the specific HbA1c targets established for patients with type 2 diabetes.^6^, ^8^ This more intensive weight management approach may help physicians and patients decide on the most effective weight loss treatments, as well as alleviate the metabolic comorbidities associated with obesity.

Greater waist circumference typically indicates the coexistence of obesity‐related diseases, and the significant reduction in waist circumference accompanying weight loss after metabolic surgery, observed in the present study, may help reduce obesity‐related complications.^13^, ^35^

Although greater TWL and reductions in BMI and waist circumference were observed after SG than after RYGB, similar percentages of patients achieved successful weight loss based on an EWL >50% and alleviation of metabolic markers at 12 months postoperatively. Though studies reported greater and more sustained weight loss after RYGB than after SG,^36^ other studies found that RYGB and SG brought about similar reductions in TWL and BMI.^37^, ^38^ Therefore, the long‐term effects of weight loss between RYGB and SG in this study population should be investigated by continued follow‐up. Although more patients with type 2 diabetes at baseline underwent RYGB, patients who underwent SG showed similar improvements in glycemic control and insulin resistance, and both surgical procedures significantly improved lipid parameters. Among patients with type 2 diabetes, the SG group had lower BMIs and waist circumference, and showed greater improvements in liver function. Those in the RYBG group had more apparent alleviation in dyslipidemia. Similar improvement of glycometabolism achieved in both surgical procedures. Among patients without type 2 diabetes, the change in body weight and BMI was similar in both procedures. Better liver function and lower blood pressure and blood glucose levels were attained by SG, whereas greater improvement in dyslipidemia was obtained by RYBG. SG and RYGB are the most widely performed metabolic surgeries, with SG increasingly performed compared with RYGB.^11^, ^37^, ^39^ Successful weight loss is achieved with both SG and RYGB; however, SG is faster and easier to perform, and the incidence of nutritional deficiency is lower after SG than after RYGB. Moreover, diseases affecting the bypassed reserved stomach developing after RYGB cannot be detected with gastroscopy, whereas narrow gastric sleeve development after SG can be detected with gastroscopy.^5^ Nevertheless, SG is associated with certain risks, including gastroesophageal reflux disease, Barrett's esophagus, and greater long‐term weight gain; thus, laparoscopic surgeons have explored modified procedures based on SG.^37^, ^40^, ^41^, ^42^

The low rate of surgical complications associated with metabolic surgery indicates the safety of SG and RYGB for patients with obesity in mainland China, as in other countries.^9^, ^10^, ^18^, ^19^, ^20^ Current metabolic surgery guidelines recommend regular postoperative follow‐up^11^, ^12^, ^13^, ^14^, ^15^, ^16^, ^17^, ^18^, ^19^, ^20^, ^21^, ^22^, ^23^, ^24^, ^25^, ^26^, ^31^; however, as observed in other studies, the follow‐up rates declined over time in the present study.^43^, ^44^ In mainland China, many patients with obesity undergo metabolic surgery to improve their physical appearance and not to alleviate their risk of various metabolic comorbidities associated with obesity per se.^9^, ^10^, ^11^, ^12^, ^13^ In this study, patients with higher BMI and obesity complications at baseline were more likely to return to the hospital 12 months postoperatively for follow‐up, indicating that the baseline condition of the patients affected follow‐up compliance; if such patients are satisfied with their physical appearance within the 12 months following surgery, they may feel that further follow‐up is unnecessary, and this may explain the lower follow‐up rate over time for patients with lower BMI and fewer obesity comorbidities. The greater reduction of hemoglobin in women than in men in both SG and RYGB groups might be related to the increased the risk of anemia during the normal menstrual cycle after operation for most women of reproductive age. In addition, previous studies have shown that patients who have undergone SYGB are more liable to have malnutrition than those who have undergone SG.^45^, ^46^ Hematological results at follow‐up can help guide nutrient supplementation to effectively reduce nutritional deficiencies; thus, patients who do not attend routine follow‐up, especially women and those who have undergone SYGB, may be at increased risk of nutritional deficiencies over time.^9^, ^10^, ^11^, ^12^, ^13^, ^18^, ^19^, ^20^ Improving follow‐up rates is a challenge for metabolic surgery centers worldwide.

The prevalence of *H. pylori* in China and worldwide was calculated to be marginally higher than 40% but was relatively lower in the present study, possibly because of the young age of the patients.^47^, ^48^

This study has several limitations. This was a retrospective observational study with a relatively short follow‐up duration. Further, remission of obesity‐related diseases, including diabetes, hypertension, and NAFLD, and administration of medicines, such as anti‐diabetic, antihypertensive, and lipid lowing drugs, were not addressed. Finally, we did not analyze quality of life parameters and those related to loss to follow‐up at 12 months. Most patients with type 2 diabetes underwent RYGB, while most subjects with simple obesity underwent SG based on the suggestions from the guidelines and consensus of metabolic surgeons.^6^, ^7^, ^8^, ^9^, ^10^ However, two of the centers in the present study preferred SG, which may have biased the comparison of the two surgical procedures. Long‐term, prospective, multicenter, and randomized controlled studies of metabolic surgery are needed to confirm our results and the beneficial effects of metabolic surgery on cardiovascular disease and death in the Chinese population with obesity.

In conclusion, for most Chinese patients with moderate to severe obesity, metabolic surgery resulted in successful weight loss and improvement in metabolic disorders at 12 months after surgery. EWL and alleviation of metabolic markers did not significantly differ between SG and RYGB.

## CONFLICT OF INTEREST STATEMENT

The authors have no conflicts of interest to declare.

## Supporting information


**Supplementary Figure S1.** Proportion of patients who returned for follow‐up.

**Supplementary Figure S2.** Proportion of patients who returned for follow‐up per hospital (A). **p < 0.01 *p < 0.05 other hospitals vs Daping Hospital, Third Military Medical University; *##p* < 0.01 #*p* < 0.05 other hospitals vs Zhongshan Hospital Affiliated with Fudan University; *&&p* < 0.01 &*p* < 0.05 other hospitals vs Drum Tower Hospital Affiliated with Nanjing University Medical School. Proportion of patients who returned for follow‐up per surgical group (B), and per sex (C). **p < 0.01 *p < 0.05. RYGB, Roux‐en‐Y gastric bypass; SG, sleeve gastrectomy.

**Supplemental Table S1.** Number of surgeons and surgical selection per hospital.

**Supplemental Table S2.** Baseline obesity‐related comorbidities of patients who returned for follow‐up.

**Supplemental Table S3.** Inflammation and nutrition biomarkers at baseline and at 12 months.

**Supplemental Table S4.** Nutrition biomarkers at baseline and at 12 months according to sex.

**Supplemental Table S5.** Nutrition biomarkers in the SG and RYBG groups at baseline and at 12 months.

**Supplemental Table S6.** Postoperative comorbidities of patients who returned for follow‐up during the 12‐month follow‐up period.

**Supplemental Table S7.** Baseline characteristics of the patients who returned for follow‐up and of those who did not.

**Supplemental Table S8.** Characteristics of the patients with type 2 diabetes in the SG and RYBG groups at baseline and at 12 months.

**Supplemental Table S9.**. Characteristics of the patients without type 2 diabetes in the SG and RYBG groups at baseline and at 12 months.

**Supplemental Table S10.** Obesity‐related comorbidities at baseline in patients who returned for follow‐up and in those who did not.
