# Supplementary figures and images for: Weight loss and metabolic benefits of bariatric surgery in China: A multicenter study
Source: J Diabetes. 2023 Jul 6;15(9):787–98. doi: 10.1111/1753-0407.13430 (PMC10509516; doi:10.1111/1753-0407.13430)

Supplemental Figure 1

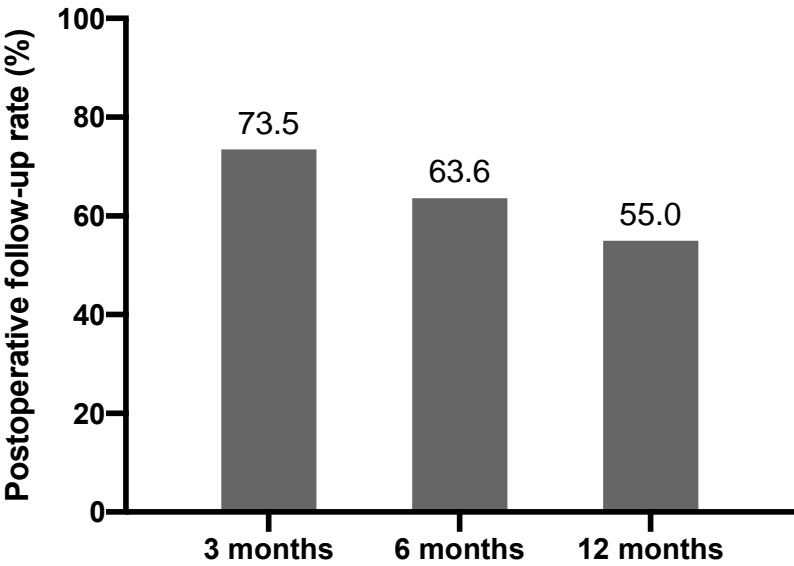

Supplement: Supplementary file 1 — Supplementary Figure S1. Proportion of patients who returned for follow‐up. [file JDB-15-787-s001.pdf]

Supplemental Figure 2

A

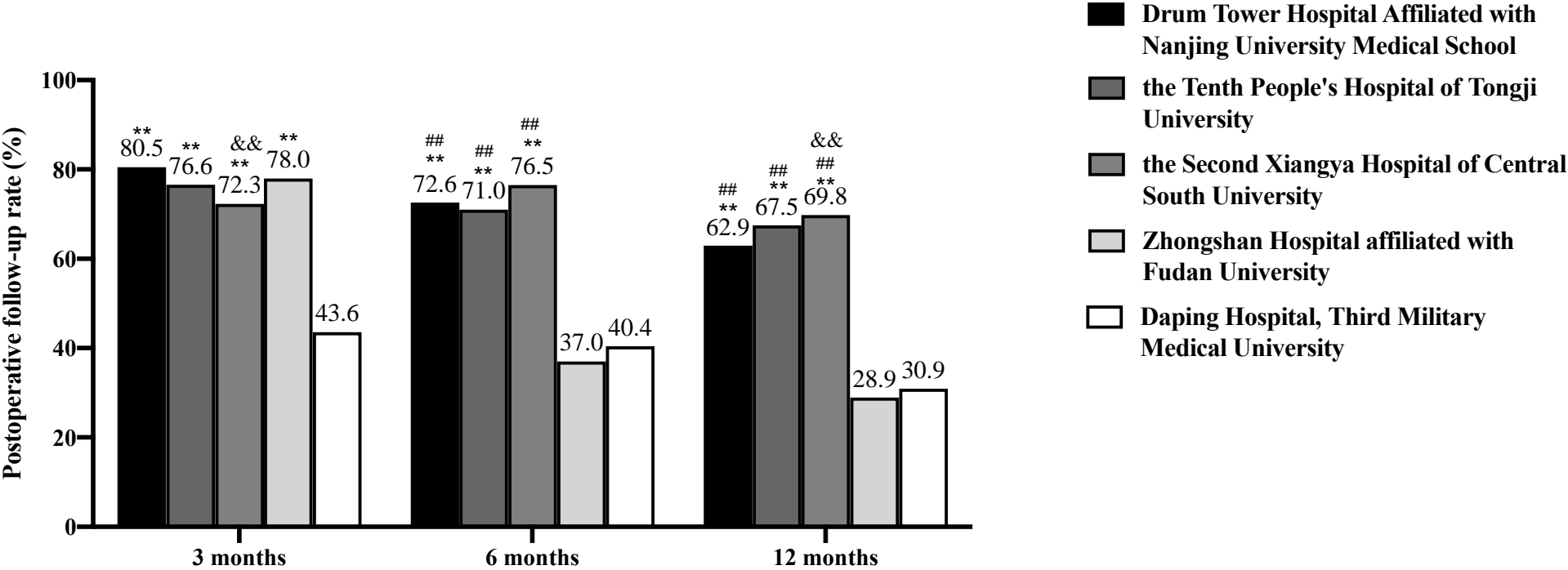

## Supplemental Figure 2

**B**

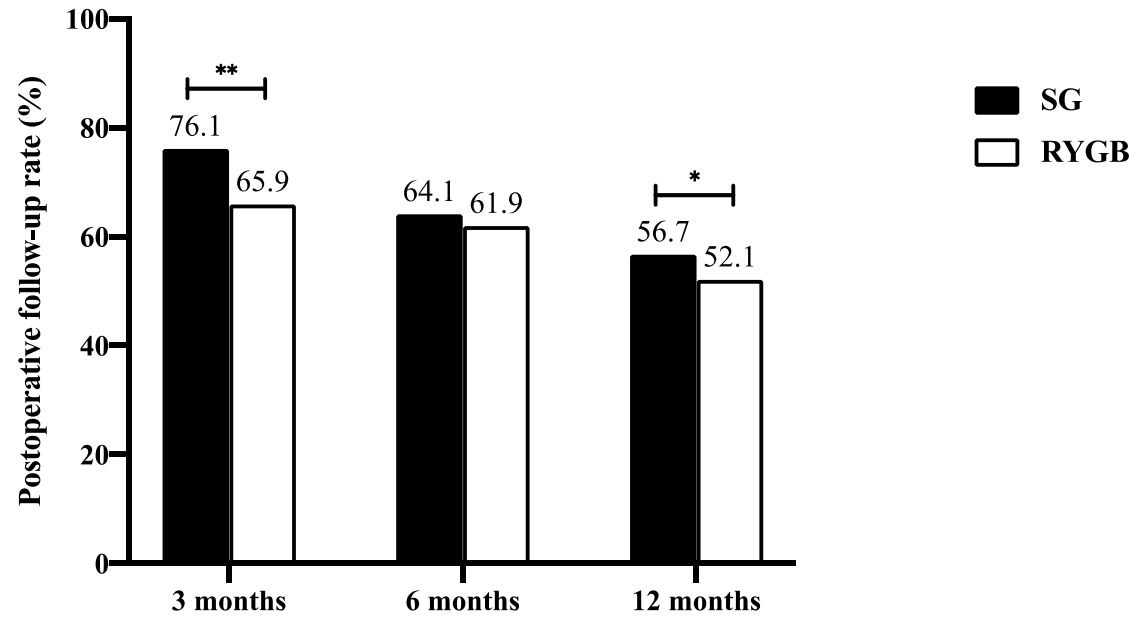

**C**

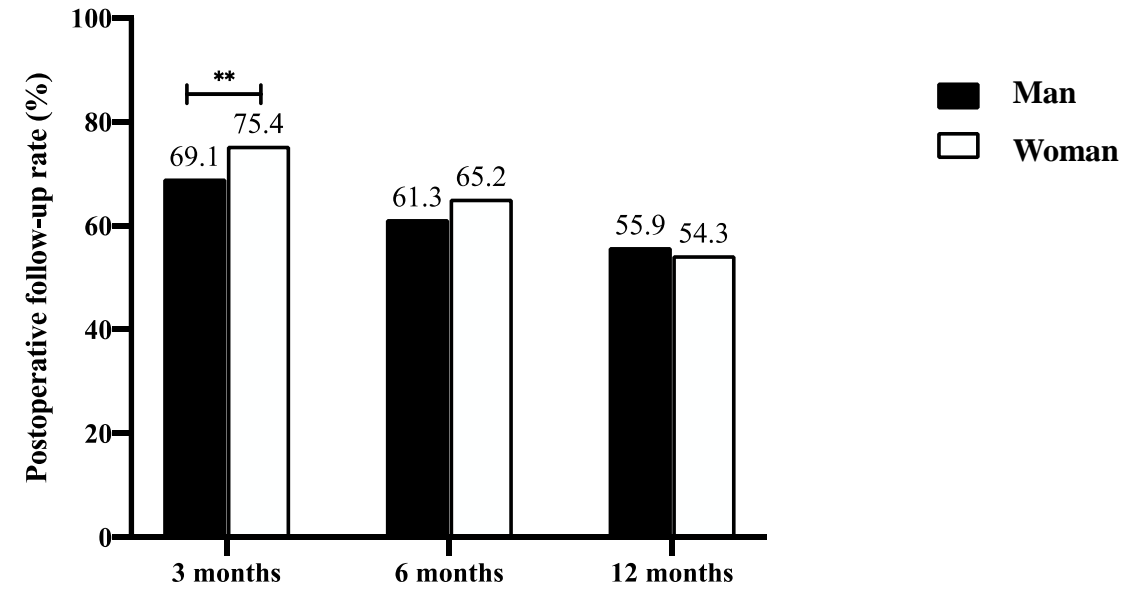

Supplement: Supplementary file 2 — Supplementary Figure S2. Proportion of patients who returned for follow‐up per hospital (A). **p < 0.01 *p < 0.05 other hospitals vs Daping Hospital, Third Military Medical University; ##p < 0.01 #p < 0.05 other hospitals vs Zhongshan Hospital Affiliated with Fudan University; &&p < 0.01 &p < 0.05 other hospitals vs Drum Tower Hospital Affiliated with Nanjing University Medical School. Proportion of patients who returned for follow‐up per surgical group (B), and per sex (C). **p < 0.01 *p < 0.05. RYGB, Roux‐en‐Y gastric bypass; SG, sleeve gastrectomy. [file JDB-15-787-s008.pdf]
