# Supplementary material for: Weight loss and metabolic benefits of bariatric surgery in China: A multicenter study
Source: J Diabetes. 2023 Jul 6;15(9):787–98. doi: 10.1111/1753-0407.13430 (PMC10509516; doi:10.1111/1753-0407.13430)
Supplement: Supplementary file 3 — Supplemental Table S1. Number of surgeons and surgical selection per hospital. [file JDB-15-787-s002.docx]

**Supplemental Table 1. Number of sugeons and surgical selection per hospital**

|  | Total | SG | RYGB |
| --- | --- | --- | --- |
| Drum Tower Hospital Affiliated with  Nanjing University Medical School |  |  |  |
| Surgeons n | 4 | - | - |
| Patients n (%) | 221 | 91 (41.2) | 130 (58.8) |
| the Tenth People's Hospital of Tongji University |  |  |  |
| Surgeons n | 6 | - | - |
| Patients n (%) | 126 | 113 (89.7) | 13 (10.3) |
| the Second Xiangya Hospital of Central South University |  |  |  |
| Surgeons n | 5 | - | - |
| Patients n (%) | 106 | 75 (70.8) | 31 (29.2) |
| Zhongshan Hospital affiliated with Fudan University |  |  |  |
| Surgeons n | 6 | - | - |
| Patients n (%) | 97 | 95 (98.0) | 2 (2.0) |
| Daping Hospital, Third Military Medical University |  |  |  |
| Surgeons n | 4 | - | - |
| Patients n (%) | 97 | 35 (36.1) | 62 (63.9) |

Abbreviations: SG, sleeve gastrectomy; RYGB Roux‐en‐Y gastric bypass.
