# Supplementary material for: Weight loss and metabolic benefits of bariatric surgery in China: A multicenter study
Source: J Diabetes. 2023 Jul 6;15(9):787–98. doi: 10.1111/1753-0407.13430 (PMC10509516; doi:10.1111/1753-0407.13430)
Supplement: Supplementary file 4 — Supplemental Table S2. Baseline obesity‐related comorbidities of patients who returned for follow‐up. [file JDB-15-787-s007.docx]

**Supplemental Table 2. Baseline obesity-related comorbidities of patients who returned for follow-up**

|  | **Total** | **SG** | **RYGB** | ***P***  **SG vs. RYGB** |
| --- | --- | --- | --- | --- |
| **Number (n)** | **356** | **213** | **143** |  |
| **Type 2 diabetes, No. (%)** | **143 (40.2)** | **78 (33.6)** | **65 (52.4)** | **0.001** |
| **Impaired glucose regulation, No. (%)** | **100 (28.1)** | **67 (28.9)** | **33 (26.6)** | **0.650** |
| **Hypertension, No. (%)** | **217 (61.0)** | **136 (58.6)** | **81 (65.3)** | **0.217** |
| **Hyperuricemia, No. (%)** | **171 (48.0)** | **117 (50.4)** | **54 (43.5)** | **0.216** |
| **Dyslipidemia, No. (%)** | **346 (97.2)** | **225 (97.0)** | **121 (97.6)** | **1.000** |
| **Gastro-esophageal reflux, No. (%)** | **15 (4.2)** | **6 (2.6)** | **9 (7.3)** | **0.037** |
| **Helicobacter pylori infection, No. (%)** | **40 (11.1)** | **25 (10.8)** | **16 (12.1)** | **0.707** |
| **Stroke, No. (%)** | **1 (0.3)** | **0 (0)** | **1 (0.8)** | **0.348** |
| **Coronal atherosclerosis heart disease, No. (%)** | **3 (0.8)** | **1 (0.4)** | **2 (1.6)** | **0.279** |
| **Heart failure, No. (%)** | **4 (1.1)** | **2 (0.9)** | **2 (1.6)** | **0.613** |
| **Limbs venous thrombosis****, No. (%)** | **1 (0.3)** | **0 (0)** | **1 (0.8)** | **0.348** |
| **Obstructive sleep apnea , No. (%)** | **58 (16.3)** | **39 (16.8)** | **19 (15.3)** | **0.717** |
| **NAFLD, No. (%)** | **194 (54.5)** | **105 (45.3)** | **89 (71.8)** | **<0.001** |

Abbreviations: SG, sleeve gastrectomy; RYGB Roux‐en‐Y gastric bypass; NAFLD, non‐alcoholic fatty liver disease. SG, sleeve gastrectomy; RYGB Roux‐en‐Y gastric bypass. *P* values of < 0.05 were considered significant.
