# Supplementary material for: Weight loss and metabolic benefits of bariatric surgery in China: A multicenter study
Source: J Diabetes. 2023 Jul 6;15(9):787–98. doi: 10.1111/1753-0407.13430 (PMC10509516; doi:10.1111/1753-0407.13430)
Supplement: Supplementary file 5 — Supplemental Table S3. Inflammation and nutrition biomarkers at baseline and at 12 months. [file JDB-15-787-s011.docx]

**Supplemental Table 3. Inflammation and nutrition biomarkers at baseline and at 12 months**

|  | **N** | **Baseline** | **12 months** | **d** | ***P*** |
| --- | --- | --- | --- | --- | --- |
| **CRP (mg/L)** | **296** | **8.3 ± 10.3** | **3.3 ± 3.6** | **5.0 ± 0.6** | **< 0.001** |
| **WBC (*10^9/L)** | **142** | **7.8 ± 2.1** | **6.1 ± 1.8** | **1.7 ± 0.2** | **< 0.001** |
| **Neutrophils (*10^9/L)** | **142** | **4.9 ± 1.8** | **3.5 ± 1.4** | **1.4 ± 0.2** | **< 0.001** |
| **[Hemoglobin](javascript:;) (g/L)** | **277** | **140.2 ± 16.8** | **135.0 ± 19.0** | **5.2 ± 0.9** | **< 0.001** |
| **Folic acid (ng/ml)** | **89** | **11.3** **± 5.4** | **13.6 ± 6.9** | **-2.3 ± 0.8** | **0.005** |
| **Vit B12 (pg/ml)** | **119** | **508.9 ± 215.9** | **419.2 ± 195.3** | **89.7 ± 25.7** | **0.001** |
| **25 hydroxyvitamin D (ng/ml)** | **152** | **19.6 ± 9.9** | **25.7 ± 15.4** | **-6.2 ± 0.8** | **< 0.001** |

Abbreviation: CRP: C-reactive protein; WBC: white blood cell. Quantitative variables are presented as the mean ± [standard](javascript:;) deviation  (SD)
