# Supplementary material for: Weight loss and metabolic benefits of bariatric surgery in China: A multicenter study
Source: J Diabetes. 2023 Jul 6;15(9):787–98. doi: 10.1111/1753-0407.13430 (PMC10509516; doi:10.1111/1753-0407.13430)
Supplement: Supplementary file 6 — Supplemental Table S4. Nutrition biomarkers at baseline and at 12 months according to sex. [file JDB-15-787-s005.docx]

**Supplemental Table 4. Nutrition biomarkers at baseline and at 12 months according to sex**

|  | **Man** | | **Woman** | | | | | ***P* baseline** | **Estimated Treatment Difference,**  **male vs. female Mean (95% CI)** | | ***P***  **Decreased**  **value between two groups** | |
| --- | --- | --- | --- | --- | --- | --- | --- | --- | --- | --- | --- | --- |
|  | **N** | **Baseline** | | **1 year** | **N** | **Baseline** | **1 year** |  | |  | |  |
| [**Hemoglobin**](javascript:;) **(g/L)** | **133** | **150.0 ± 12.8** | | **147.9 ± 14.0** | **144** | **130.8 ± 14.8** | **122.9 ± 14.7**** | **0.000** | **-5.7 (-9.4 to -2.1)** | | | **0.002** |
| **Folic acid (ng/ml)** | **44** | **9.5 ± 4.9** | | **12.9 ± 7.3**** | **45** | **13.1 ± 5.2** | **14.2 ± 6.5** | **0.001** | **-2.4 (-5.4 to 0.7)** | | | **0.131** |
| **Vit B12 (pg/ml)** | **52** | **476.7 ± 202.9** | | **433.2 ± 228.2** | **67** | **533.9 ± 223.8** | **408.2 ± 166.3**** | **0.153** | **-82.2 (-184.2 to 19.8)** | | | **0.113** |
| **25 hydroxyvitamin D (ng/ml)** | **64** | **19.6 ± 9.5** | | **26.0 ± 14.1**** | **88** | **19.5 ± 10.2** | **25.5 ± 16.3**** | **0.949** | **-0.4 (-3.5 to 2.8)** | | | **0.810** |

***P* < 0.01 **P* < 0.05 baseline vs. 1 year. *P* values of < 0.5 were considered significant. Quantitative variables are presented as the mean ± [standard](javascript:;) deviation (SD)
