# Supplementary material for: Weight loss and metabolic benefits of bariatric surgery in China: A multicenter study
Source: J Diabetes. 2023 Jul 6;15(9):787–98. doi: 10.1111/1753-0407.13430 (PMC10509516; doi:10.1111/1753-0407.13430)
Supplement: Supplementary file 7 — Supplemental Table S5. Nutrition biomarkers in the SG and RYBG groups at baseline and at 12 months. [file JDB-15-787-s009.docx]

**Supplemental Table 5. Nutrition biomarkers in the SG and RYBG groups at baseline and at 12 months**

|  | **SG** | | **RYGB** | | | | | ***P* baseline** | **Estimated Treatment Difference,**  **SG vs. RYGB Mean (95% CI)** | | ***P***  **Decreased**  **value between two groups** |
| --- | --- | --- | --- | --- | --- | --- | --- | --- | --- | --- | --- |
|  | **N** | **Baseline** | | **1 year** | **N** | **Baseline** | **1 year** |  | |  |  |
| **Hemoglobin (g/L)** | **179** | **139.9 ± 16.5** | | **133.9 ± 18.9**** | **98** | **140.3 ± 17.5** | **136.7 ± 19.2*** | **0.841** | **2.4 (-1.4 to 6.3)** | | **0.217** |
| **Folic acid (ng/ml)** | **46** | **10.5 ± 5.0** | | **10.5 ± 6.4** | **43** | **12.2 ± 5.7** | **16.9 ± 5.8**** | **0.127** | **4.7 (1.7 to 7.6)** | | **0.002** |
| **Vit B12 (pg/ml)** | **53** | **511.8 ± 269.2** | | **492.6 ± 226.7** | **66** | **506.6 ± 163.3** | **360.1 ± 142.0**** | **0.902** | **-127.3 (-227.5 to -27.1)** | | **0.013** |
| **25 hydroxyvitamin D (ng/ml)** | **88** | **23.6 ± 9.7** | | **32.1 ± 15.9**** | **109** | **14.1 ± 7.1** | **17.0 ± 9.0**** | **0.000** | **-5.6 (-8.4 to -2.8)** | | **0.000** |

Abbreviations: SG: sleeve gastrectomy; RYGB: laparoscopy Roux‐en‐Y gastric bypass. ***P* < 0.01 **P* < 0.05 baseline vs. 1 year. *P* values of < 0.5 were considered significant. Quantitative variables are presented as the mean ± [standard](javascript:;) deviation (SD)
