# Supplementary material for: Weight loss and metabolic benefits of bariatric surgery in China: A multicenter study
Source: J Diabetes. 2023 Jul 6;15(9):787–98. doi: 10.1111/1753-0407.13430 (PMC10509516; doi:10.1111/1753-0407.13430)
Supplement: Supplementary file 8 — Supplemental Table S6. Postoperative comorbidities of patients who returned for follow‐up during the 12‐month follow‐up period. [file JDB-15-787-s006.docx]

**Supplemental Table 6. Postoperative comorbidities of patients who returned for follow up during the 12-month follow up period**

|  | **SG** | **RYGB** | ***P*** |
| --- | --- | --- | --- |
| **Anastomotic haemorrhage, %** | **0** | **0** | **-** |
| **Anastomotic stenosis, %** | **0** | **0.8** | **0.460** |
| **Anastomotic fistula, %** | **0** | **0** | **-** |
| **Wound infection, %** | **0** | **0** | **-** |
| **Internal hernia, %** | **0** | **0.8** | **0.460** |
| **Gastro-oesophageal reflux, %** | **0.7** | **0.8** | **1.000** |
| **Intestinal obstruction, %** | **0** | **0.8** | **0.460** |
| **Gastroparesis, %** | **0** | **0** | **-** |
| **Dumping syndrome, %** | **0** | **4.1** | **0.020** |
| **Cholecystitis, %** | **6.3** | **0** | **0.004** |
| **Pneumonia, %** | **0** | **0** | **-** |
| **Pulmonary embolism, %** | **0** | **0** | **-** |
| **Lithangiuria, %** | **0** | **0** | **-** |
| **Vitamin D deficiency, %** | **49.3** | **57.4** | **0.368** |
| **Bone mass decrease, %** | **4.2** | **4.9** | **0.778** |
| **Osteoporosis, %** | **0** | **0** | **-** |
| **Anaemia, %** | **8.4** | **7.4** | **0.761** |

Abbreviations: SG, sleeve gastrectomy; RYGB Roux‐en‐Y gastric bypass; Adverse events of patients in the Tenth People's Hospital of Tongji University were not included. *P*values of < 0.05 were considered significant
