# Supplementary material for: Weight loss and metabolic benefits of bariatric surgery in China: A multicenter study
Source: J Diabetes. 2023 Jul 6;15(9):787–98. doi: 10.1111/1753-0407.13430 (PMC10509516; doi:10.1111/1753-0407.13430)
Supplement: Supplementary file 9 — Supplemental Table S7. Baseline characteristics of the patients who returned for follow‐up and of those who did not. [file JDB-15-787-s003.docx]

**Supplemental Table 7. Baseline characteristics of the patients who returned for follow-up and of those who did not**

|  | **Follow-up** | **No follow-up** | ***P*** |
| --- | --- | --- | --- |
| **Number (n)** | **356** | **291** | **-** |
| **Age (years)** | **34.3 ± 0.6** | **33.9 ± 0.7** | **0.702** |
| **Sex (Man/Woman)** | **167/189** | **131/159** | **0.547** |
| **Weight (kg)** | **111.3 ± 1.3** | **106.2 ± 1.5** | **0.010** |
| **BMI (kg/m^2^)** | **39.****4** **± 0.4** | **37.6 ± 0.4** | **0.001** |
| **BMI < 24kg/m²** | **0%** | **0%** | **-** |
| **Waist circumference (cm)** | **119.****6 ± 1.0** | **116.7 ± 1.0** | **0.047** |
| **SBP (mmHg)** | **135.9 ± 1.3** | **134.8 ± 1.0** | **0.536** |
| **SBP < 130mmHg** | **40.6%** | **38.8%** | **0.687** |
| **DBP (mmHg)** | **85.2 ± 0.9** | **83.5 ± 0.7** | **0.165** |
| **HbA1c (%)** | **6.8 ± 0.1** | **6.7 ± 0.1** | **0.425** |
| **HbA1c < 7%** | **65.9%** | **67.4%** | **0.704** |
| **HbA1c < 6%** | **44.8%** | **44.3%** | **0.906** |
| **FBG (mmol/L)** | **7.****0 ± 0.2** | **6.6 ± 0.2** | **0.104** |
| **FBG < 5.6mmol/L** | **41.7%** | **46.0%** | **0.296** |
| **120 min glucose (mmol/L)** | **10.9 ± 0.3** | **11.9 ± 0.8** | **0.238** |
| **Fasting insulin (μU/mL)** | **30.9 ± 1.3** | **28.5 ± 2.1** | **0.331** |
| **120 min insulin (μU/mL)** | **144.6 ± 8.5** | **137.4 ± 7.1** | **0.513** |
| **HOMA-IR (mmol/L, IU/mL)** | **9.8 ± 0.6** | **8.1 ± 0.7** | **0.072** |
| **HOMA-IR < 1.45** | **0.4%** | **3.0%** | **0.068** |
| **TG (mmol/L)** | **2.3 ± 0.1** | **4.2 ± 0.4** | **0.000** |
| **TC (mmol/L)** | **4.6 ± 0.1** | **4.6 ± 0.1** | **0.338** |
| **HDL-C (mmol/L)** | **1.0 ± 0.0** | **1.0 ± 0.0** | **0.738** |
| **LDL-C (mmol/L)** | **2.8 ± 0.0** | **2.8 ± 0.0** | **0.580** |
| **LDL-C < 2.6mmol/L** | **42.4%** | **44.0%** | **0.691** |
| **ALT (U/L)** | **57.9 ± 2.7** | **51.6 ± 2.6** | **0.100** |
| **AST (U/L)** | **38.5 ± 2.2** | **35.3 ± 2.2** | **0.303** |
| **rGT (U/L)** | **51.0 ± 2.3** | **53.1 ± 2.7** | **0.561** |
| **Cr (umol/L)** | **60.0 ± 1.0** | **61.1 ± 0.9** | **0.376** |
| **UA (umol/L)** | **419.9 ± 5.7** | **428.1 ± 6.5** | **0.337** |

Abbreviations: SG: sleeve gastrectomy; RYGB laparoscopy Roux‐en‐Y gastric bypass; BMI: body mass index; SBP: systolic blood pressure; DBP: diastolic blood pressure; HbA1c: glycated hemoglobin; FBG: fasting blood glucose; HOMA-IR: homeostasis model assessment of insulin resistance; TG: triglycerides; TC; total lipoprotein cholesterol; HDL-C: high-density lipoprotein cholesterol; LDL-C: low-density lipoprotein cholesterol; ALT: serum alanine aminotransferase; AST: aspartate aminotransferase; GGT: glutamyltrans peptidase; Cr: creatinine UA: uric acid.
