# Supplementary material for: Weight loss and metabolic benefits of bariatric surgery in China: A multicenter study
Source: J Diabetes. 2023 Jul 6;15(9):787–98. doi: 10.1111/1753-0407.13430 (PMC10509516; doi:10.1111/1753-0407.13430)
Supplement: Supplementary file 10 — Supplemental Table S8. Characteristics of the patients with type 2 diabetes in the SG and RYBG groups at baseline and at 12 months. [file JDB-15-787-s012.docx]

**Supplement Table 8. Characteristics of the patients with type 2 diabetes in the SG and RYBG groups at baseline and at 12 months**

|  | **SG** | | **RYGB** | | | | | | ***P* baseline** | | **Estimated Treatment Difference, SG vs. RYGB Mean (95% CI)** | | ***P***  **Decreased value between two groups** | | |
| --- | --- | --- | --- | --- | --- | --- | --- | --- | --- | --- | --- | --- | --- | --- | --- |
|  | **N** | **Baseline** | | **1 year** | **N** | **Baseline** | **1 year** |  | | | |  | | |  |
| **Number (n)** | **-** | **78** | | **-** | **-** | **65** | **-** | **--** | | **-** | | | | **-** | |
| **Sex (Man/Woman)** | **-** | **37/41** | | **-** | **-** | **33/32** | **--** | **0.819** | | **-** | | | | **-** | |
| **Age (years)** | **-** | **35.2 ± 11.5** | | **-** | **-** | **40.3 ± 11.3** | **-** | **0.009** | | **-** | | | | **-** | |
| **Weight (kg)** | **-** | **107.8 ± 19.7** | | **76.0 ± 15.0**** | **-** | **104.2 ± 20.4** | **78.5 ± 14.3**** | **0.285** | | **-4.7（-7.5 to -1.8)** | | | | **0.002** | |
| **BMI (kg/m^2^)** | **-** | **38.3 ± 5.2** | | **27.0 ± 4.1**** | **-** | **37.9 ± 7.3** | **28.5 ± 4.4**** | **0.746** | | **-1.7 (-2.6 to -0.7)** | | | | **0.001** | |
| **BMI < 24kg/m²** | **-** | **0%** | | **25.6%** | **-** | **0%** | **13.8%** | **1.000** | | **-** | | | | **0.124** | |
| **Waist circumference (cm)** | **70** | **118.9 ± 12.0** | | **91.8 ± 11.5**** | **64** | **117.4 ± 17.3** | **97.0 ± 12.5**** | **0.563** | | **-6.7 (-9.8 to -3.7)** | | | | **0.000** | |
| **SBP (mmHg)** | **77** | **134.7 ± 18.8** | | **119.2 ± 17.5** | **61** | **143.3 ± 25.5** | **121.4 ± 17.0** | **0.025** | | **-0.3 (-7.7 to 7.2)** | | | | **0.942** | |
| **SBP < 130mmHg** | **-** | **51.1%** | | **72.3%** | **-** | **31.3%** | **68.8%** | **0.130** | | **-** | | | | **0.926** | |
| **DBP (mmHg)** | **77** | **84.6 ± 14.4** | | **75.2 ± 13.2** | **61** | **89.5 ± 15.7** | **74.6 ± 10.4** | **0.063** | | **1.1 (-4.4 to 6.5)** | | | | **0.698** | |
| **HbA1c (%)** | **78** | **7.2 ± 1.8** | | **5.4 ± 0.6** | **63** | **8.7 ± 1.9** | **5.7 ± 0.7** | **0.000** | | **-0.2 (-0.5 to 0.0)** | | | | **0.063** | |
| **HbA1c < 7%** | **-** | **54.4%** | | **97.1%** | **-** | **15.1%** | **98.1%** | **0.000** | | **-** | | | | **1.000** | |
| **HbA1c < 6%** | **-** | **39.7%** | | **91.2%*** | **-** | **37.7%** | **75.5%*** | **0.000** | | **-** | | | | **0.035** | |
| **FBG (mmol/L)** | **78** | **8.8 ± 2.7** | | **4.8 ± 0.7**** | **65** | **9.9 ± 3.4** | **5.4 ± 1.2**** | **0.032** | | **-0.6 (-0.9 to -0.2)** | | | | **0.001** | |
| **FBG < 5.6mmol/L** | **-** | **5.4%** | | **9.5%**** | **-** | **1.7%** | **33.3%**** | **0.379** | | **-** | | | | **0.001** | |
| **120 min glucose (mmol/L)** | **78** | **15.1 ± 3.5** | | **5.0 ± 2.3*** | **63** | **16.3 ± 4.5** | **5.9 ± 2.8*** | **0.078** | | **-1.0 (-1.9 to -0.0)** | | | | **0.040** | |
| **Fasting insulin (uU/mL)** | **61** | **35.5 ± 26.6** | | **10.2 ± 6.0** | **48** | **25.3 ± 21.0** | **11.0 ± 7.9** | **0.031** | | **-1.7 (-4.4 to 1.0)** | | | | **0.202** | |
| **120 min insulin (uU/mL)** | **60** | **153.9 ± 15.6** | | **31.0 ± 38.6** | **48** | **75.8 ± 70.5** | **21.2 ± 19.5** | **0.000** | | **10.1 (-5.4 to 25.5)** | | | | **0.197** | |
| **HOMA-IR (mmol/L,uU/mL)** | **57** | **14.1 ± 12.5** | | **2.2 ± 1.3** | **41** | **12.3 ± 13.4** | **2.8 ± 2.9** | **0.478** | | **-0.7 (-1.5 to 0.2)** | | | | **0.126** | |
| **HOMA-IR < 1.45** | **-** | **0%** | | **36.8%** | **-** | **0%** | **29.3%** | **1.000** | | **-** | | | | **0.571** | |
| **TG (mmol/L)** | **78** | **2.3 ± 1.3** | | **1.0 ± 0.4** | **64** | **3.5 ± 3.1** | **1.1 ± 0.4** | **0.003** | | **-0.1 (-0.2 to 0.1)** | | | | **0.317** | |
| **TC (mmol/L)** | **78** | **4.5 ± 1.0** | | **4.5 ± 0.9**** | **64** | **5.1 ± 1.3** | **4.2 ± 0.9**** | **0.002** | | **0.6 (0.4 to 0.9)** | | | | **0.000** | |
| **HDL-C (mmol/L)** | **78** | **1.0 ± 0.3** | | **1.4 ± 0.3** | **64** | **1.0 ± 0.3** | **1.3 ± 0.4** | **0.724** | | **0.1 (-0.1 to 0.2)** | | | | **0.350** | |
| **LDL-C (mmol/L)** | **78** | **2.7 ± 0.9** | | **2.7 ± 0.9**** | **64** | **2.9 ± 1.0** | **2.3 ± 0.7**** | **0.267** | | **0.5 (0.2 to 0.7)** | | | | **0.000** | |
| **LDL-C < 2.6mmol/L** | **-** | **47.2%** | | **45.8%*** | **-** | **44.1%** | **69.5%*** | **0.854** | | **-** | | | | **0.011** | |
| **ALT (U/L)** | **78** | **60.6 ± 46.9** | | **14.7 ± 12.9**** | **64** | **48.3 ± 42.8** | **26.3 ± 11.2**** | **0.108** | | **-12.2 (-16.5 to -8.0)** | | | | **0.000** | |
| **AST (U/L)** | **78** | **37.4 ± 25.2** | | **17.0 ± 7.4**** | **64** | **43.7 ± 69.6** | **23.8 ± 10.8**** | **0.464** | | **-6.7 (-10.0 to -3.3)** | | | | **0.000** | |
| **GGT (U/L)** | **72** | **52.8 ± 35.3** | | **16.6 ± 13.1** | **45** | **55.3 ± 42.4** | **22.2 ± 15.8** | **0.732** | | **-4.4 (-9.4 to 0.5)** | | | | **0.079** | |
| **Cr (umol/L)** | **75** | **59.2 ± 15.0** | | **60.5 ± 12.6*** | **64** | **60.7 ± 21.4** | **59.3 ± 15.8*** | **0.631** | | **2.9 (0.0 to 5.8)** | | | | **0.048** | |
| **UA (umol/L)** | **78** | **406.7 ± 93.4** | | **345.5 ± 91.0** | **65** | **390.8 ± 109.4** | **350.8 ± 95.8** | **0.350** | | **-10.0 (-36.4 to 16.3)** | | | | **0.453** | |

Abbreviations: SG: sleeve gastrectomy; RYGB: laparoscopy Roux‐en‐Y gastric bypass; BMI: body mass index; SBP: systolic blood pressure; DBP: diastolic blood pressure; HbA1c: glycated hemoglobin; FBG: fasting blood glucose; HOMA-IR: homeostasis model assessment of insulin resistance; TG: triglycerides; TC; total lipoprotein cholesterol; HDL-C: high-density lipoprotein cholesterol; LDL-C: low-density lipoprotein cholesterol; ALT: serum alanine aminotransferase; AST: aspartate aminotransferase; GGT: glutamyltrans peptidase; Cr: creatinine UA: uric acid.

***P* < 0.01 **P* < 0.05 baseline vs. 1 year. *P* values of < 0.5 were considered significant. Quantitative variables are presented as the mean ± [standard](javascript:;) deviation (SD)
