# Supplementary material for: Weight loss and metabolic benefits of bariatric surgery in China: A multicenter study
Source: J Diabetes. 2023 Jul 6;15(9):787–98. doi: 10.1111/1753-0407.13430 (PMC10509516; doi:10.1111/1753-0407.13430)
Supplement: Supplementary file 11 — Supplemental Table S9.. Characteristics of the patients without type 2 diabetes in the SG and RYBG groups at baseline and at 12 months. [file JDB-15-787-s010.docx]

**Supplement Table 9. Characteristics of the patients without type 2 diabetes in the SG and RYBG groups at baseline and at 12 months**

|  | **SG** | | **RYGB** | | | | | | ***P* baseline** | | **Estimated Treatment Difference, SG vs. RYGB Mean (95% CI)** | | ***P***  **Decreased value between two groups** | | |
| --- | --- | --- | --- | --- | --- | --- | --- | --- | --- | --- | --- | --- | --- | --- | --- |
|  | **N** | **Baseline** | | **1 year** | **N** | **Baseline** | **1 year** |  | | | |  | | |  |
| **Number (n)** | **-** | **154** | | **-** | **-** | **59** | **-** | **-** | | **-** | | | | **-** | |
| **Sex (Man/Womam)** | **-** | **76/78** | | **-** | **-** | **21/38** | **-** | **0.099** | | **-** | | | | **-** | |
| **Age (years)** | **-** | **31.4 ± 10.4** | | **-** | **-** | **33.9 ± 9.4** | **-** | **0.114** | | **-** | | | | **-** | |
| **Weight (kg)** | **-** | **115.5 ± 25.8** | | **79.1 ± 15.9** | **-** | **112.5 ± 25.1** | **78.4 ± 16.4** | **0.445** | | **-0.9 (-3.6 to 1.9)** | | | | **0.546** | |
| **BMI (kg/m^2^)** | **-** | **40.2 ± 7.4** | | **27.3± 4.5** | **-** | **40.5 ± 6.9** | **28.2 ± 4.5** | **0.778** | | **-0.5 (-1.5 to 0.4)** | | | | **0.249** | |
| **BMI < 24kg/m²** | **-** | **0%** | | **24.0%** | **-** | **0%** | **18.6%** | **1.000** | | **-** | | | | **0.510** | |
| **Waist circumference (cm)** | **132** | **120.1 ± 16.6** | | **92.3 ± 11.4** | **52** | **120.9 ± 15.3** | **92.3 ± 14.1** | **0.780** | | **1.9 (-2.3 to 4.9)** | | | | **0.491** | |
| **SBP (mmHg)** | **152** | **137.0 ± 17.4** | | **119.0 ± 14.0** | **55** | **135.0 ± 15.6** | **120.3 ± 15.7** | **0.450** | | **-2.1 (-7.7 to 3.4)** | | | | **0.443** | |
| **SBP < 130mmHg** | **-** | **39.1%** | | **79.0%** | **-** | **39.3%** | **75.0%** | **1.000** | | **-** | | | | **0.839** | |
| **DBP (mmHg)** | **152** | **85.1 ± 13.4** | | **74.0 ± 10.8*** | **55** | **86.7 ± 13.9** | **79.1 ± 12.0*** | **0.441** | | **-4.6 (-9.0 to -0.2)** | | | | **0.040** | |
| **HbA1c (%)** | **150** | **6.1 ± 1.2** | | **5.2 ± 0.4** | **55** | **6.3 ± 1.6** | **5.3 ±0.4** | **0.309** | | **-0.1 (-0.2 to -0.1)** | | | | **0.271** | |
| **HbA1c < 7%** | **-** | **83.7%** | | **99.2%** | **-** | **89.8%** | **100%** | **0.431** | | **-** | | | | **1.000** | |
| **HbA1c < 6%** | **-** | **61.2%** | | **96.1%** | **-** | **53.1%** | **93.9%** | **0.412** | | **-** | | | | **0.809** | |
| **FBG (mmol/L)** | **153** | **5.3 ± 0.7** | | **4.4 ± 0.5**** | **56** | **5.4 ± 0.7** | **4.7 ± 0.5**** | **0.122** | | **-0.2 (-0.4 to -0.1)** | | | | **0.005** | |
| **FBG < 5.6mmol/L** | **-** | **69.1%** | | **97.1%** | **-** | **68.6%** | **94.1%** | **1.000** | | **-** | | | | **0.609** | |
| **120 min glucose (mmol/L)** | **152** | **7.4 ± 1.9** | | **4.4 ± 1.3** | **55** | **7.8 ± 1.7** | **4.4 ± 1.0** | **0.214** | | **0.04 (-0.4 to 0.5)** | | | | **0.849** | |
| **Fasting insulin (uU/mL)** | **111** | **28.5 ± 17.0** | | **9.2 ± 5.2** | **51** | **30.9 ± 16.6** | **9.6 ± 3.6** | **0.412** | | **-0.5 (-2.2 to 1.2)** | | | | **0.548** | |
| **120 min insulin (uU/mL)** | **110** | **151.2 ± 122.9** | | **34.0 ± 53.7*** | **51** | **145.9 ± 89.4** | **12.8 ± 17.8*** | **0.783** | | **22.0 (3.9 to 40.1)** | | | | **0.018** | |
| **HOMA-IR (mmol/L,uU/mL)** | **90** | **7.3 ± 4.3** | | **1.9 ± 1.2** | **44** | **7.0 ± 3.7** | **2.0 ± 0.8** | **0.743** | | **-0.1 (-0.5 to 0.3)** | | | | **0.513** | |
| **HOMA-IR < 1.45** | **-** | **1.1%** | | **41.1%** | **-** | **0%** | **27.3%** | **1.000** | | **-** | | | | **0.170** | |
| **TG (mmol/L)** | **150** | **1.9 ± 1.3** | | **1.5 ± 6.5** | **56** | **1.8 ± 0.9** | **1.0 ± 0.5** | **0.471** | | **0.5 (-1.3 to 2.3)** | | | | **0.619** | |
| **TC (mmol/L)** | **149** | **4.6 ± 0.9** | | **4.4 ± 0.9**** | **55** | **4.5 ± 0.8** | **4.0 ± 0.7**** | **0.444** | | **0.4 (0.1 to 0.6)** | | | | **0.005** | |
| **HDL-C (mmol/L)** | **149** | **1.0 ± 0.2** | | **1.4 ± 0.3** | **56** | **1.1 ± 0.6** | **1.4 ± 0.3** | **0.030** | | **0.0 (-0.0to 0.1)** | | | | **0.317** | |
| **LDL-C (mmol/L)** | **151** | **2.9 ± 0.7** | | **2.6 ± 0.8**** | **55** | **2.6 ± 0.7** | **2.2 ± 0.6**** | **0.016** | | **0.4 (0.1 to 0.6)** | | | | **0.006** | |
| **LDL-C < 2.6mmol/L** | **-** | **36.8%** | | **55.6%** | **-** | **48%** | **72.0%** | **0.229** | | **-** | | | | **0.065** | |
| **ALT (U/L)** | **150** | **60.4 ± 49.4** | | **13.7 ± 7.8**** | **58** | **56.8 ± 46.9** | **22.2 ± 11.6**** | **0.633** | | **-8.6 (-11.5 to -5.6)** | | | | **0.000** | |
| **AST (U/L)** | **149** | **61.6 ± 15.4** | | **16.2 ± 5.6**** | **54** | **57.8 ± 12.3** | **20.2 ± 6.0**** | **0.513** | | **-4.0 (-5.9 to -2.1)** | | | | **0.000** | |
| **GGT (U/L)** | **141** | **52.5 ± 50.9** | | **17.2 ± 10.4** | **48** | **45.8 ± 27.1** | **18.1 ± 8.5** | **0.387** | | **-1.6 (-4.6 to 1.3)** | | | | **0.268** | |
| **Cr (umol/L)** | **149** | **61.6 ± 15.4** | | **59.9 ± 16.0** | **54** | **57.8 ± 12.3** | **54.8 ± 10.7** | **0.070** | | **2.3 (-0.7 to 5.3)** | | | | **0.124** | |
| **UA (umol/L)** | **151** | **441.1 ± 100.4** | | **355.7 ± 92.2*** | **57** | **425.9 ± 102.0** | **324.6 ±73.7*** | **0.332** | | **23.6 (0.8 to 46.5)** | | | | **0.043** | |

Abbreviations: SG: sleeve gastrectomy; RYGB: laparoscopy Roux‐en‐Y gastric bypass; BMI: body mass index; SBP: systolic blood pressure; DBP: diastolic blood pressure; HbA1c: glycated hemoglobin; FBG: fasting blood glucose; HOMA-IR: homeostasis model assessment of insulin resistance; TG: triglycerides; TC; total lipoprotein cholesterol; HDL-C: high-density lipoprotein cholesterol; LDL-C: low-density lipoprotein cholesterol; ALT: serum alanine aminotransferase; AST: aspartate aminotransferase; GGT: glutamyltrans peptidase; Cr: creatinine UA: uric acid.

***P* < 0.01 **P* < 0.05 baseline vs. 1 year. *P* values of < 0.5 were considered significant. Quantitative variables are presented as the mean ± [standard](javascript:;) deviation (SD)
