# Supplementary material for: Weight loss and metabolic benefits of bariatric surgery in China: A multicenter study
Source: J Diabetes. 2023 Jul 6;15(9):787–98. doi: 10.1111/1753-0407.13430 (PMC10509516; doi:10.1111/1753-0407.13430)
Supplement: Supplementary file 12 — Supplemental Table S10. Obesity‐related comorbidities at baseline in patients who returned for follow‐up and in those who did not. [file JDB-15-787-s004.docx]

**Supplemental Table 10. Obesity-related comorbidities at baseline in patients who returned for follow up and in those who did not**

|  | **Follow-up** | **No follow-up** | ***P*** |
| --- | --- | --- | --- |
| **Number (n)** | **356** | **291** |  |
| **Type 2 diabetes, No. (%)** | **143 (40.2)** | **124 (42.6)** | **0.530** |
| **Impaired glucose regulation, No. (%)** | **100 (28.1)** | **57 (19.6)** | **0.012** |
| **Hypertension, No. (%)** | **217 (61.0)** | **116 (39.9)** | **0.000** |
| **Hyperuricemia, No. (%)** | **171 (48.0)** | **141 (48.5)** | **0.915** |
| **Dyslipidemia, No. (%)** | **346 (97.2)** | **256 (88.0)** | **0.000** |
| **Gastro-esophageal reflux, No. (%)** | **15 (4.2)** | **7 (2.4)** | **0.207** |
| **Helicobacter pylori infection, No. (%)** | **41 (11.5)** | **7 (2.4)** | **0.000** |
| **Stroke, No. (%)** | **1 (0.3)** | **1 (0.3)** | **1.000** |
| **Coronal atherosclerosis heart disease, No. (%)** | **3 (0.8)** | **1 (0.3)** | **0.631** |
| **Heart failure, No. (%)** | **4 (1.1)** | **1 (0.3)** | **0.386** |
| **Limbs venous thrombosis****, No. (%)** | **1 (0.3)** | **1 (0.3)** | **1.000** |
| **Obstructive sleep apnea , No. (%)** | **58 (16.3)** | **50 (17.2)** | **0.763** |
| **NAFLD, No. (%)** | **194 (54.5)** | **135 (46.4)** | **0.040** |

Abbreviations: NAFLD, non‐alcoholic fatty liver disease. *P* values of < 0.05 were considered significant.
